# Surgical Repair of Posterobasal Ventricular Septal Rupture Complicated by Severe Tricuspid Regurgitation: A Case Report

**DOI:** 10.70352/scrj.cr.25-0728

**Published:** 2026-04-01

**Authors:** Masaru Yoshikai, Hisashi Sato, Nagi Hayashi, Kouta Shimauchi

**Affiliations:** Department of Cardiovascular Surgery, Shin-Koga Hospital, Kurume, Fukuoka, Japan

**Keywords:** posterobasal ventricular septal rupture, tricuspid regurgitation, valve replacement, right atrial approach, right ventricular infarction

## Abstract

**INTRODUCTION:**

Posterobasal ventricular septal rupture (P-VSR) developed after acute myocardial infarction (AMI) is often associated with right ventricular infarction and carries a high surgical mortality. The coexistence of tricuspid regurgitation (TR) further aggravates right ventricular dysfunction and right heart failure. Therefore, surgical repair of P-VSR complicated by right ventricular infarction and severe TR is particularly challenging.

**CASE PRESENTATION:**

A 75-year-old woman developed acute inferior myocardial infarction complicated by right ventricular infarction. A coronary stent was deployed for right coronary artery occlusion. On day 16 after the onset of AMI, transthoracic echocardiography revealed a P-VSR and severe TR, and she was transferred to our institution for surgical management. Through a right atrial approach, excellent visualization of the P-VSR was obtained. The septal defect was closed securely using two bovine pericardial patches placed on both the right and left ventricular sides of the ventricular septum. Tricuspid valve replacement (TVR) was also performed. Postoperative echocardiography confirmed complete closure of the defect without residual shunt.

**CONCLUSIONS:**

In the surgical treatment of P-VSR complicated by severe TR, a right atrial approach may provide adequate exposure of the septal defect while potentially minimizing additional ventricular injury. In selected patients with severe TR in a similar anatomical and clinical context, concomitant TVR may represent a feasible surgical option.

## Abbreviations

AMI
: acute myocardial infarction
ECG
: electrocardiogram
EF
: ejection fraction
LV
: left ventricle
MR
: mitral regurgitation
PA
: pulmonary artery
P-VSR
: posterobasal ventricular septal rupture
Qp/Qs
: pulmonary-to-systemic flow ratio
RA
: right atrium
RCA
: right coronary artery
RV
: right ventricle
TR
: tricuspid regurgitation
TTE
: transthoracic echocardiography
TVR
: tricuspid valve replacement
VSR
: ventricular septal rupture

## INTRODUCTION

P-VSR developed after AMI is frequently associated with RV infarction and carries a high surgical mortality.^1)^ Although the coexistence of TR with VSR is rare,^2,3)^ TR increases RV volume overload, further deteriorating RV function and exacerbating right heart failure. Therefore, precise management of TR is essential during surgical repair. Surgical treatment of P-VSR complicated by RV infarction and severe TR is extremely challenging. We present a case of successful repair using an RA approach for P-VSR closure combined with TVR.

## CASE PRESENTATION

A 75-year-old woman with a history of hypertension, dyslipidemia, and diabetes mellitus presented to a clinic with resting dyspnea and chest tightness; however, her ECG showed no abnormalities. Nine days later, she was admitted to another hospital with recurrent dyspnea and chest tightness at rest. On admission, ECG demonstrated ST-segment elevation in leads II, III, aVF, and V4R. TTE revealed akinesis of the inferior wall. Coronary angiography revealed occlusion in segment 2 of the RCA, leading to a diagnosis of acute inferior and RV myocardial infarction. A coronary stent was deployed. Heart failure persisted despite medical therapy, and continuous catecholamine support was required to maintain hemodynamics. On day 16 after admission, TTE revealed shunt flow from the LV to the RV suggestive of a VSR in the inferobasal septal wall, accompanied by severe TR. Cardiac catheterization demonstrated an oxygen step-up between the vena cavae and the RA, and between the RA and RV. Hemodynamic measurements were as follows: mean RA pressure 15 mmHg, RV pressure 63/19 mmHg, PA pressure 47/19 mmHg (mean 32 mmHg), mean PA wedge pressure 38 mmHg, Qp/Qs 5.6, and left-to-right shunt ratio 82%, confirming the diagnosis of P-VSR. The patient was transferred to our institution on day 17 after the onset of AMI for surgical treatment.

**Fig. 1:**
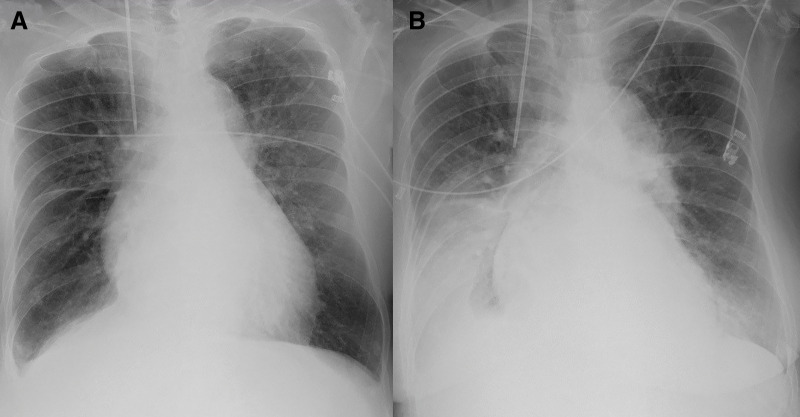
Chest radiograph findings. Chest radiograph obtained on day 5 after transfer (**B**) shows worsening pulmonary congestion compared with that taken at the time of transfer (**A**).

**Fig. 2:**
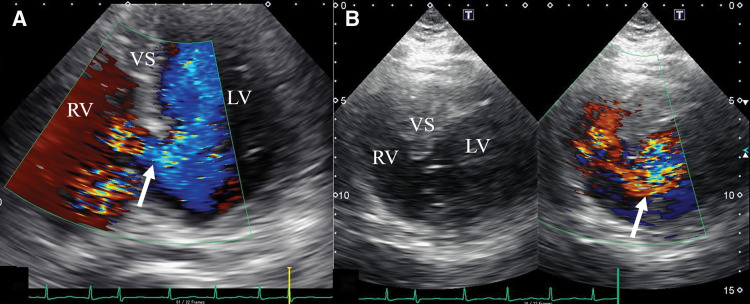
Transthoracic echocardiographic findings. Apical (**A**) and short-axis (**B**) views show a left-to-right shunt jet (arrows) from the left ventricle to the right ventricle. LV, left ventricle; RV, right ventricle; VS, ventricular septum

**Fig. 3:**
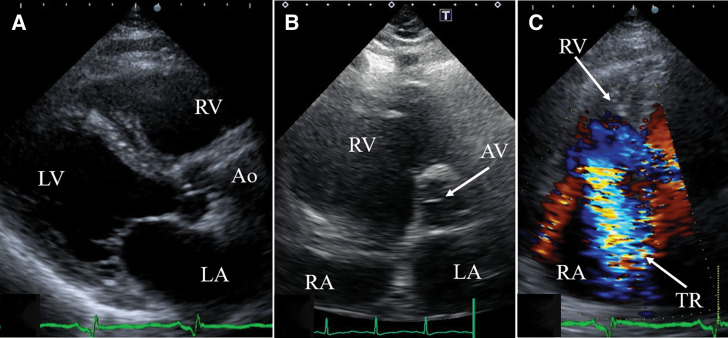
Transthoracic echocardiographic findings. Parasternal long-axis view (**A**) and short-axis view (**B**) demonstrate right ventricular enlargement. The apical view (**C**) demonstrates severe tricuspid regurgitation. Ao, aorta; AV, aortic valve; LA, left atrium; LV, left ventricle; RA; right atrium; RV, right ventricle; TR, tricuspid regurgitation

ECG at transfer showed Q waves and inverted T waves in leads II, III, and aVF. Chest radiograph demonstrated a cardiothoracic ratio of 58% and slight pulmonary congestion (**Fig. 1A**). TTE revealed a LV end-diastolic/end-systolic diameter of 48/28 mm, EF of 70%, estimated systolic PA pressure of 56 mmHg, a 14-mm VSR at the inferobasal septal wall (**Fig. 2**), and enlargement of both atria and the RV. The calculated Qp/Qs was 2.5, with severe TR (**Fig. 3**), mild-to-moderate MR, and an estimated mean RA pressure of 15 mmHg. Because the timing of VSR onset was uncertain and the patient was hemodynamically stable, conservative therapy was initially continued after transfer. However, progressive heart failure developed (**Fig. 1B**), and estimated systolic PA pressure elevated to 69 mmHg; therefore, surgical intervention was performed on day 23 after AMI.

**Fig. 4:**
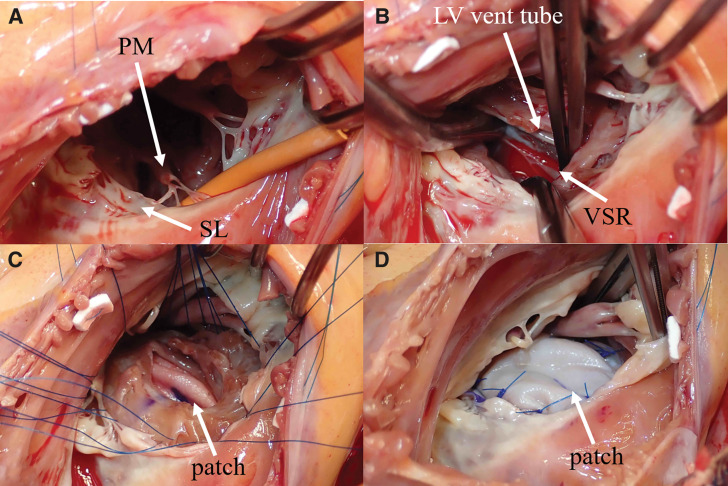
Operative findings. A rubber-covered probe was inserted into the ventricular septal rupture, and this papillary muscle was transected (**A**). A left ventricular vent tube is observed through the VSR (**B**). A patch was inserted into the left ventricular side using full-thickness sutures on the ventricular septum (**C**). The procedure to close the ventricular septal rupture has been completed, and the patch on the right ventricular side can be demonstrated (**D**). LV, left ventricle; PM, papillary muscle; SL, septal leaflet of the tricuspid valve; VSR, ventricular septal rupture

Intraoperative transesophageal echocardiography under general anesthesia showed moderate MR. After establishing cardiopulmonary bypass with ascending aortic and bicaval cannulations, systemic cooling was initiated, and ventricular fibrillation was induced. Right atriotomy and right-sided left atriotomy were performed. A 15 × 15-mm VSR was identified approximately 1 cm away from the tricuspid annulus, accompanied by annular dilation. The mitral valve exhibited posterior leaflet shortening and anterior leaflet thickening with calcification, rendering valve replacement necessary. After cardioplegic arrest, the VSR was found to be located on the tricuspid annular side of the papillary muscle supporting the septal–posterior commissure (**Fig. 4A**), and this papillary muscle was transected (**Fig. 4B**), which provided an excellent surgical view of the VSR. The septal defect was repaired by sandwiching the ventricular septum between two bovine pericardial patches placed on the LV (**Fig. 4C**) and RV sides (**Fig. 4D**) using transmural sutures passed from the LV side through the septum to the RV side. Mitral valve replacement with preservation of subvalvular structures and TVR using bioprostheses was then completed. Weaning from cardiopulmonary bypass was uneventful. Postoperatively, the patient required nitric oxide therapy for hypoxemia and was extubated on POD 4. POD 7 TTE demonstrated no residual shunt and good prosthetic valve function, although the LVEF decreased to approximately 30%. She was transferred for rehabilitation on POD 21. At 8 months after surgery, TTE showed recovery of LVEF to 56%, with localized akinesis at the inferobasal wall. At the 5-year follow-up TTE, inferobasal wall akinesis persisted; however, indices of RV function were preserved, with tricuspid annular plane systolic excursion of 18.3 mm, RV fractional area change of 44.6%, and S’ velocity of 11 cm/s. The patient has remained well and active for 5 years after surgery without recurrence of heart failure.

## DISCUSSION

P-VSR develops secondary to AMI involving occlusion of the RCA or the left circumflex artery. When caused by RCA occlusion, it is frequently accompanied by RV infarction and is associated with high surgical mortality.^1)^ Although the coexistence of TR with VSR is uncommon,^2)^ TR may result from papillary muscle rupture,^3,4)^ annular dilation, or RV dysfunction,^5)^ thereby exacerbating right heart failure. Recent studies have reported that even mild or greater TR at the onset of AMI increases the long-term risk of major adverse cardiac events.^6)^ In general, the surgical goals in VSR repair are to achieve complete closure of the septal defect without residual shunting and to avoid additional impairment of LV and RV function. Therefore, in cases of P-VSR complicated by severe TR, precise control of TR is of particular importance, in addition to ensuring complete closure of the septal defect and avoiding further impairment of LV and RV function. In the present case, no rupture of the tricuspid papillary muscles was observed. The TR was likely caused by annular dilation and RV enlargement with leaflet tethering. During the procedure, a single papillary muscle was transected, allowing exceptional visualization of the VSR and facilitating its precise closure. Repair of either the leaflets or the transected papillary muscle, combined with implantation of an annuloplasty ring, was considered; however, because ensuring reliable control of TR was of paramount importance, TVR was performed. The necessity of mitral valve surgery in the present case remains controversial. In patients with postinfarction VSR, moderate or greater MR has been shown to be a risk factor for operative mortality.^2)^ In this case, structural abnormalities of the mitral valve itself were identified, and a reduction in MR could not be expected after surgery. Therefore, mitral valve replacement was performed.

Several surgical approaches have been described for P-VSR, including LV approach,^7–9)^ RV approach,^10)^ and RA approaches.^5,11,12)^

The LV approach involves elevating the cardiac apex and incising the LV parallel to the posterior interventricular sulcus.^7,9)^ The space between the ventricular septum and the posteromedial papillary muscle of the mitral valve is narrow, and there is a risk of injuring this papillary muscle during the LV incision, which results in MR. Moreover, incising and suturing the fragile LV wall can lead to bleeding from the suture line or further impair LV function. When performing VSR repair using the infarct-exclusion technique,^8,9)^ the presence of the posteromedial papillary muscle makes the procedure technically demanding. Moreover, this procedure may alter LV geometry and affect the mitral papillary muscles. In addition, if the patch is undersized, it can result in low output syndrome or diastolic dysfunction. Therefore, we do not recommend the LV approach for the surgical repair of P-VSR.

RV approach^10)^ involves elevating the apex and incising the RV along the posterior interventricular sulcus, followed by patch closure of the septal defect. Kinoshita et al. applied an extended sandwich patch technique via RV incision in 33 patients, including 12 with posterior VSR, reporting a 30-day mortality of 18% (6/33) and only one patient requiring reoperation for residual shunt within 1 month.^10)^ They noted that although it remains unclear whether tricuspid annular plane systolic excursion and RV fractional area change can accurately assess RV dysfunction caused by RV incision, these parameters did not change before and after surgery. This approach can indeed avoid altering the LV geometry and is considered to carry a lower risk of bleeding. However, in patients with RV infarction and right heart failure, right ventriculotomy may further impair RV function, and the incision site may eventually form a scar and serve as a substrate for ventricular arrhythmia in the long term.^12)^ Moreover, concomitant tricuspid valve procedures cannot be performed in the same operative field, which seems to be another limitation.^12)^

RA approach^5,11,12)^ avoids incision into infarcted ventricles, minimizing the risk of bleeding, and allows secure VSR closure comparable to the RV extended sandwich patch technique.^10)^ The RA approach seems to provide better visualization of the defect than the RV approach, permitting direct observation of the mitral valve chordae and papillary muscles and facilitating secure transmural sutures with full-thickness bites. Evaluation of the VSR location using TTE and transesophageal echocardiography, together with intraoperative direct echocardiography, is useful for determining whether the RA approach is feasible. When the VSR is located close to the tricuspid annulus, the RA approach is considered achievable. Ultimately, the ability to clearly visualize and safely place sutures along the apical margin of the VSR from the RA is critical. Because the RV is not incised, RV function is preserved, making the RA approach the safest and most effective method for P-VSR. However, if the VSR lies on the tricuspid annular side of the papillary muscles supporting the septal leaflet or septal–posterior commissure, detachment of these papillary muscles or leaflets may be necessary to obtain adequate exposure.^12)^ Numerous studies have demonstrated that division of the mitral chordae tendineae impairs LV function,^13,14)^ whereas evidence regarding the impact of tricuspid papillary muscle division on RV function remains limited. RV systolic function is determined by three principal mechanisms: (1) inward movement of the free wall, producing a bellows effect; (2) contraction of longitudinal fibers, resulting in shortening of the long axis and apical displacement of the tricuspid annulus; and (3) traction on the free wall at the sites of attachment secondary to LV contraction.^15)^ The tricuspid subvalvular apparatus contributes primarily to longitudinal contraction. In the present case, the divided papillary muscle originated from the ventricular septum and supported the septal–posterior commissure; given its small size and short distance to the tricuspid annulus, its impact on RV function was considered minimal. Compared with the adverse impact of severing the mitral subvalvular apparatus on LV function, transection of a single papillary muscle of the tricuspid valve is expected to have minimal effect on RV function.

In the present case, transection of a single papillary muscle allowed excellent exposure of the VSR. Under such circumstances, either tricuspid valve repair^12,16)^ or TVR^5)^ is required. The 2020 American College of Cardiology/American Heart Association guidelines for TR state that TVR may be required in the presence of severe annular dilatation or intrinsic leaflet pathology to prevent residual or recurrent TR, although no definitive recommendation is provided regarding repair versus replacement.^17)^ Surgical series have reported superior long-term survival with tricuspid valve repair compared with TVR; however, these findings are limited by baseline imbalances and nonrandomized designs.^18)^ In contrast, a propensity score–matched analysis demonstrated comparable mid-term survival between repair and replacement, suggesting that TVR should not be avoided when there is concern for recurrent TR after repair.^19)^ Preoperative isolated RV dilatation^20)^ and higher TR grade^21)^ are predictors of recurrent TR and early repair failure. In addition, residual moderate-to-severe or greater TR after transcatheter tricuspid valve repair is associated with reduced 2-year survival.^22)^ In the present case, tricuspid valve repair would have required artificial chordal reconstruction of the leaflet supported by the divided papillary muscle in addition to annuloplasty. However, myocardial infarction involved not only the ventricular septum but also the RV free wall; consequently, no reliable non-infarcted papillary muscle suitable for anchoring artificial chordae was identified. Taken together with the presence of RV dilatation, annular enlargement, tricuspid leaflet tethering, and division of one papillary muscle, the risk of residual or recurrent TR after repair was considered high. In surgery for VSR associated with RV infarction and severe TR, residual TR in the acute postoperative phase would exacerbate RV volume overload. Therefore, to avoid this adverse condition and to ensure secure elimination of TR, TVR was selected in this case. Based on these considerations, this case suggests that concomitant TVR may be considered in selected patients with P-VSR complicated by severe TR, particularly when adequate control of regurgitation is unlikely to be achieved by repair alone.

## CONCLUSIONS

In the surgical treatment of P-VSR complicated by severe TR, an RA approach may provide adequate exposure of the septal defect while potentially minimizing additional ventricular injury. In selected patients with severe TR in a similar anatomical and clinical context, concomitant TVR may represent a feasible surgical option.

## DECLARATIONS

### Availability of data and materials

Data are available on request to the corresponding author.
